# Barriers to Treatment and Control of Hypertension among Hypertensive Participants: A Community-Based Cross-sectional Mixed Method Study in Municipalities of Kathmandu, Nepal

**DOI:** 10.3389/fcvm.2016.00026

**Published:** 2016-08-02

**Authors:** Surya Devkota, Raja Ram Dhungana, Achyut Raj Pandey, Bihungum Bista, Savyata Panthi, Kartikesh Kumar Thakur, Ratna Mani Gajurel

**Affiliations:** ^1^Manmohan Cardiothoracic, Vascular and Transplant Centre, Institute of Medicine, Tribhuvan University, Kathmandu, Nepal; ^2^Nepal Family Development Foundation, Kathmandu, Nepal; ^3^Nepal Health Research Council, Kathmandu, Nepal; ^4^Nature Care Hospital, Kathmandu, Nepal

**Keywords:** hypertension, awareness, treatment, control, barriers, Nepal

## Abstract

**Introduction:**

Despite the established evidence on benefits of controlling raised blood pressure and development of several guidelines on detection and management of hypertension, people often have untreated or uncontrolled hypertension. In this context, we undertook this study to identify the barriers existing in hypertension treatment and control in the municipalities of Kathmandu district in Nepal.

**Methods:**

This was a community based, cross-sectional mixed method study conducted in the municipalities of Kathmandu district in Nepal between January and July 2015. Among 587 randomly selected participants, the aware hypertensive participants were further assessed for the treatment and control of hypertension. For qualitative component, 20 participants having uncontrolled hypertension took part in two focused group discussions and two cardiac physicians participated in in-depth interviews.

**Results:**

Out of 587 participants screened, 191 (32.5%) were identified as hypertensive. Among 191 hypertensive participants, 118 (61.8%) were aware of their problem. Of the 118 aware hypertensive participants, 93 (78.8%) were taking medicines, and among those treated, 46 (49.6%) had controlled hypertension. Proportions of participants taking anti-hypertensive medications varied significantly with age groups, ethnicity, occupation and income. Hypertension control was significantly associated with use of combination therapy, adherence to medication, follow-up care, counseling by health-care providers, and waiting time in hospital. Being worried that the medicine needs to be taken lifelong, perceived side effects of drugs, non-adherence to medication, lost to follow-up, inadequate counseling from physician, and lack of national guidelines for hypertension treatment were the most commonly cited barriers for treatment and control of hypertension in qualitative component of the research.

**Conclusion:**

Large proportion of the hypertensive population has the untreated and the uncontrolled hypertension. Efforts to dispel and dismantle the myths and barriers related to hypertension treatment and control are warranted to reduce the consequences of uncontrolled hypertension.

## Introduction

Hypertension is one of the most common causes of cardiovascular disease (CVD) and deaths globally (1). A relatively small increase in blood pressure affects the incidence of various CVD including coronary artery disease, congestive heart failure, renal insufficiency, peripheral vascular disease, and stroke (2, 3).

Despite the evidence on benefits of lowering blood pressure, and the existence of several guidelines on detection and management of hypertension, untreated and uncontrolled hypertension occur largely in the society (4, 5). Literatures suggest that nearly half of aware hypertensive cases do not take treatment and more than half of those treated have uncontrolled blood pressure (4, 5).

Several factors relating to patients, physician, and health-care system are responsible for poor treatment and suboptimal control of hypertension (6, 7). Lack of adequate treatment, reluctance to change the lifestyle, failure to take medications as indicated, non-adherence to medicine, lack of follow-up, perceived side effect of drug, therapeutic inertia, communication barriers between health-care providers and recipient, non-compliance to treatment guidelines, and lack of access to health care are some of the mostly cited impediments for hypertension treatment and control (6–16). However, there is the dearth of similar studies in Nepal. We undertook this study to identify the barriers existing in hypertension treatment and control in the newly declared municipalities of Kathmandu.

## Materials and Methods

### Study Design, Settings, and Participants

This was a community-based, cross-sectional study conducted in the newly declared (declared in December 2014) municipalities of Kathmandu district, Nepal, between January and July 2015. The 10 municipalities, which contain more than 300,000 population, are located on the outskirts of Kathmandu metropolitan city (17).

Total 587 persons having age between 18 and 70 years and registered as permanent residents of the study areas at the time of study were selected by multistage stratified sampling method and screened for hypertension. The selection process has been described in details elsewhere (18). Among the persons screened, the aware hypertensive participants were included in the quantitative part of the study. For qualitative method, 20 participants, who had uncontrolled hypertension at the time of interview and were not included in quantitative component, were selected for two focused group discussions (FGD). Similarly, two cardiac physicians from tertiary hospital took part in in-depth interview (IDI).

### Data Collection

Quantitative data were collected *via* face-to-face interview, anthropometric measurements, and clinical examinations. Fifteen medical professionals were recruited and trained in data collection methods. Investigators themselves were involved in conducting FGDs and IDIs for qualitative data collection.

#### Face-to-face Interview

The survey questionnaire (derived from WHO STEPwise questionnaires) covered the demographics and health behaviors of respondents (19). Demographic information included age, sex, ethnicity, marital status, years at school, and primary occupation. The health behaviors included tobacco use, alcohol consumption, fruit and vegetable consumption, and physical activity. In addition, questionnaires related to knowledge and perception of hypertension; awareness, treatment, and control of hypertension; antihypertensive medication; adherence; health-care facility; and health-care providers were also included in survey tool.

##### Tobacco Use and Alcohol Consumption

Questions were asked to determine the current users of tobacco and alcohol.

##### Diet

Seventy-two hour dietary recall was used to estimate fruit and vegetable consumption. Determination of the amount of fruit and vegetables consumed was aided by pictorial show cards and standard measuring cups.

##### Physical Activity

Seven day history of physical activity was recorded. The physical activity related to work, transport, and recreational activities were categorized into vigorous, moderate, and low levels of activity in accordance with published guidelines (20, 21). Vigorous physical activity was defined as any activity that had more than six Metabolic Equivalent of Tasks (MET). Moderate physical activity was the activity that had MET values between three and six (20). Physical activity having less than three METs, like spending time sitting at a desk, traveling in a car, bus or train, etc., was considered low or sedentary physical activity.

##### Diabetes

Participants were requested to provide information on diabetic status or medication history.

##### Knowledge and Perception of Hypertension

Knowledge-based questions recorded the respondent’s understanding on high blood pressure. Perception entailed the perceived severity, chronicity, complication, and manageability of hypertension.

##### Awareness, Treatment, and Control of Hypertension

Participants were asked whether they were aware of the raised blood pressure they had. Information regarding type and name of currently used antihypertensive drugs was also obtained. Reasons behind the stoppage of or not initiating the antihypertensive medicine were collected from the hypertensive respondents who were not in treatment.

##### Adherence

Morisky four-Item Self-Report MEASURE of Medication-taking Behavior tool was used to assess the adherence of antihypertensive medication among participants who were on treatment. It contained four questions relating to forgetfulness to take the medicine (two questions) and self-adjustment of medicine dosages (two questions) – with options “Yes,” “No” in reply. The “Yes” rated answers were assigned a value of 1, and “No” as 0. Then, the aggregate number was categorized as 0 for high adherence, 1 to 2 for medium adherence, and 3 to 4 for low adherence (22, 23).

##### Health-care Facility and Health-care Providers

Health-care facility and health-care provider-related questionnaires were designed to assess the level of access to health facility, waiting time, types of health facility, types of health-care providers, communication with health-care providers on lifestyle modification, and satisfaction to service provider.

#### Anthropometric Measurement

Height and weight were measured using the digital weighing machines and the portable standard stature scales, respectively, from which body mass index (BMI) was calculated. Waist and hip circumferences were also measured by non-stretchable measuring tape in order to determine the waist–hip ratio.

#### Clinical Examination

Trained data collectors measured blood pressure using an aneroid sphygmomanometer. Before taking the measurements, participants were requested to sit quietly and rest for 15 min with legs uncrossed. Three readings of the systolic and diastolic blood pressure were taken with 3-min rest between each reading, and the mean of second and third reading was used for analysis.

#### Focused Group Discussion

Focus group discussion was the key method to explain the patient-related and system-related barriers in hypertension treatment and control. Questions were asked to hypertensive participants who had uncontrolled blood pressure. FGD guidelines consisted of knowledge and perception of hypertension; barriers in treatment and control of hypertension; antihypertensive medication; adherence; follow-up; health-care facility; and health-care providers.

#### In-depth Interview

In-depth interview was used to explore the problems and interpret issues in multidimensional ways. Two cardiac physicians were asked about the health system (human work force and other resources) and health-care provider-related barriers (adherence to clinical guidelines, advice for lifestyle modifications).

#### Operational Definitions

Standard operational definitions were adopted for key variables to maintain consistency and uniformity of the information.

##### Aware Hypertensive

A person was defined as aware if he/she was hypertensive and self-reported the condition (5).

##### Treatment

Treatment is defined as having a history of current antihypertensive drug therapy at the time of the interview (24).

##### Controlled Blood Pressure

Control of hypertension was defined as blood pressure below 140/90 mmHg while taking treatment (5).

##### Poor

Those participants having income less than 40,933 Nepalese Rupees per annum were considered the poor (25).

##### Current Smoker

Current smokers were defined as those who reported smoking any tobacco product within last 30 days.

##### Current Alcohol Drinkers

Those who consumed alcohol within last 30 days were considered current alcohol drinkers (26).

##### Sufficient Fruit and Vegetables Intake

Intake of at least 400 g of fruit and vegetables in a day was regarded as sufficient (27).

##### Sufficient Physical Activity

Sufficient physical activity was defined as ≥600 METs of moderate and vigorous activity in a week (20).

##### Hypertension

The diagnostic criterion for hypertension was set as a systolic blood pressure ≥140 mmHg and/or a diastolic blood pressure ≥90 mmHg as recommended by Joint National Committee-VII. The persons who were using antihypertensive medicine were also considered as hypertensive (28).

##### Diabetes

Self-reported use of any anti-diabetic medication was the diagnostic criteria for diabetes.

### Validity and Reliability

Prior using the tools, pilot testing survey was conducted among 20 participants, and changes were made in the data collection tools accordingly. Data enumerators were trained in data collection methods to standardize the data collection procedure and maintain the data quality. Operational definitions were followed as per the WHO, international and national guidelines. To collect valid and reliable data, investigators, themselves, conducted FGD and IDI.

### Ethical Consideration

The study protocol was reviewed and approved by Ethical Review Board of the Nepal Health Research Council, Kathmandu. Written consent was obtained from all participants after detailed explanation of research purpose and assurance of maintaining privacy and confidentiality. Those who needed further treatment were referred to tertiary treatment centers.

### Data Management and Analysis

Data were compiled, edited, and checked to maintain consistency. Duplications were removed before coding and entering them in Epidata V.2.1. Data were then exported to SPSS V.16.0 for analysis.

Frequencies and percentages were calculated to identify the distribution of sociodemographic characteristics. Chi-square test was conducted for comparing proportions of categorical variables with respect to treatment and control of hypertension. All tests were two-tailed, and *p* < 0.05 was considered statistically significant.

Qualitative data were analyzed using thematic analysis method. Information was coded with keyword approach, categorized, and finalized with the appropriate theme. Researchers independently read the transcripts line by line and remained neutral to code the documents. In second stage, open codes were merged together to develop the theme.

## Results

### Sociodemographic Characteristics

Out of 587 participants screened, 191 were identified as hypertensives. Among 191 hypertensive participants, 118 were aware of their condition. Aware hypertensive participants included 64 (54.2%) females and 54 (45.8%) males. The majority of aware hypertensive participants (59.3%) had age between 40 and 60 years. More than half of the participants (56.8%) had either no formal education or had attained primary and lower than primary education. Levels of education and types of occupation varied significantly with gender (*p* < 0.001) (Table 1).

**Table 1:** **Sociodemographic characteristics by gender among aware hypertensive participants**.

Characteristics	Categories	Gender		Total	*P* value
		Male (%)	Female (%)		
Age groups	<40 years	5 (9.3)	8 (12.5)	13 (11.0)	0.67
	40–59 years	31 (57.4)	39 (60.9)	70 (59.3)	
	≥60 years	18 (33.3)	17 (26.6)	35 (29.7)	
Education	No formal education	8 (14.8)	12 (25.0)	48 (40.7)	<0.01
	Primary and lower	8 (14.8)	40 (62.5)	19 (16.1)	
	Secondary	26 (48.1)	11 (17.2)	35 (29.7)	
	Bachelor and higher	12 (22.2)	9 (14.1)	16 (13.6)	
Ethnicity	Brahman	18 (33.3)	21 (32.8)	39 (33.1)	0.38
	Chhetri	15 (27.8)	19 (29.7)	34 (28.8)	
	Newar	15 (27.8)	11 (17.2)	26 (22.0)	
	Others (Dalit, Janajati)	6 (11.1)	13 (20.3)	19 (16.1)	
Occupation	Job	15 (27.8)	2 (3.1)	17 (14.4)	<0.01
	Self employed	15 (27.8)	4 (6.2)	19 (16.1)	
	Homemaker	8 (14.8)	53 (82.8)	61 (51.7)	
	Others (unemployed, retired)	16 (29.6)	5 (7.8)	21 (17.8)	
Poverty	Above poverty line	31 (57.4)	32 (48.4)	62 (52.5)	0.04
	Below poverty line	23 (42.6)	33 (51.6)	56 (47.5)	

*Primary and lower education, grade 1–5; secondary, grade 6–12; Job, governmental or non-governmental employment; self-employed, working for oneself as a freelancer or the owner of a business or working in own farm; homemaker, a person, especially a housewife, who manages a home; other occupation, volunteer or student or unemployed or retired; below poverty line, income less than 40,933 Nepalese rupees per annum*.

Twenty participants, equal numbers of male and female, took part in two separate focused grouped discussions. All participants had age more than 40 years. The majority of participants (70%) had less than secondary education. More than three quarters (78%) of participants belonged to Brahmin and Chhetri ethnic groups. Two key informants, cardiology physicians from a tertiary care center, provided in-depth information on patient-related and physician-related barriers in treatment and control of hypertension.

### Treatment of Hypertension

#### Sociodemographic Characteristics and Treatment of Hypertension

Among 118 aware hypertensive participants, 93 (78.8%) were taking medicines for hypertension. The majority of the participants under medication, who visited other than government hospitals (71%), had their health checkup done by specialist physician (58.0%) and were using calcium channel blockers (53.8%) for the control of hypertension. Two participants had been to primary health-care centers for the treatment of hypertension. The remaining participants (25 out of 118) had either not started (16 out of 25) or discontinued (9 out of 25) medication.

Almost equal proportions of male and female were on anti-hypertensive treatment. However, proportions of participants taking anti-hypertensive medications varied significantly with age groups, ethnicity, occupation, and income (Table 2). There was no statistically significant difference in treatment status by education levels.

**Table 2:** **Factors associated with treatment and control of hypertension among aware (*N*_1_ = 118) and treated (*N*_2_ = 93) hypertensive participants**.

Characteristics	Categories	*N*_1_	Treatment of hypertension among aware hypertensives *n*_1_ (%)	*P* value	*N*_2_	Control of hypertension among treated hypertensives *n*_2_ (%)	*P* value
Total		118	93 (78.8)		93	46 (49.6)	
Gender	Male	54	42 (77.8)	0.48	42	20 (47.6)	0.77
	Female	64	51 (79.7)		51	26 (51.0)	
Age groups	<40 years	13	7 (58.8)	<0.01	7	5 (71.4)	0.48
	40–59 years	70	53 (75.7)		53	12 (12.9)	
	≥60 years	35	33 (94.3)		33	16 (48.5)	
Education	No formal education	48	36 (75.0)	0.76	36	20 (55.6)	0.08
	Primary and lower	19	15 (78.9)		15	4 (26.7)	
	Secondary	35	28 (80.0)		28	12 (42.9)	
	Bachelor and higher	16	14 (87.5)		14	10 (71.4)	
Ethnicity	Brahman	39	36 (92.3)	<0.01	36	19 (52.8)	0.65
	Chhetri	34	27 (79.4)		27	15 (56.6)	
	Newar	26	20 (76.9)		20	8 (40.0)	
	Others (Dalit, Janjati)	19	10 (52.6)		10	4 (40.0)	
Occupation	Government job	17	12 (70.6)	<0.01	12	8 (66.7)	0.54
	Self employed	19	10 (52.6)		10	5 (50.0)	
	Homemaker	61	51 (83.6)		51	25 (49.0)	
	Others	21	20 (95.2)		20	8 (40.0)	
Poverty	Above poverty line	62	54 (87.1)	0.04	39	15 (38.5)	0.07
	Below poverty line	56	39 (69.6)		54	31 (57.4)	
Smoking	Yes	28	17 (60.7)	0.01	17	8 (47.1)	0.83
	No	90	76 (84.4)		76	38 (50.0)	
Alcohol consumption	Yes	32	21 (65.6)	0.03	21	9 (42.9)	0.49
	No	86	72 (83.7)		72	37 (51.4)	
Fruit and vegetables intake	Sufficient	15	13 (86.7)	0.42	13	3 (23.1)	0.08
	Non-sufficient	103	80 (77.7)		80	43 (53.8)	
Physical activity	Sufficient	85	64 (75.3)	0.13	64	34 (53.1)	0.30
	Non-sufficient	33	29 (87.9)		29	12 (41.4)	
BMI	≥25 kg/m^2^	74	56 (75.7)	0.28	56	29 (51.8)	0.58
	<25 kg/m^2^	44	37 (84.1)		37	17 (45.9)	
Diabetes	Yes	23	20 (87.0)	0.29	20	11 (55.0)	0.58
	No	95	73 (76.8)		73	35 (47.9)	

*N_1_, aware hypertensive participants; N_2_, aware hypertensive participants who were under treatment; n_1_, proportions of participants who were under treatment; n_2_, proportions of participants having controlled blood pressure; primary and lower education, grade 1–5; secondary, grade 6–12; job, governmental or non-governmental employment; self-employed, working for oneself as a freelancer or the owner of a business or working in own farm; Homemaker, a person, especially a housewife, who manages a home; other occupation, volunteer or student or unemployed or retired; Below poverty line, income less than 40,933 Nepalese rupees per annum; sufficient fruit and vegetable consumption, intake of ≥400 g of fruit and vegetable per day; Sufficient physical activity, ≥600 METs of moderate and vigorous activity in a week*.

#### Knowledge, Perception, and Treatment of Hypertension

More than three quarters (98 of 118) of aware hypertensive participants perceived hypertension as severe health condition. Similarly, 79.6% (94 of 118) of participants agreed that hypertension could have complications in future if left untreated. The majority of the respondents (72 of 118) also believed that hypertension was a chronic condition, whereas almost all (107 of 118) thought hypertension could be managed by lifestyle modification. However, participants’ perceptions on severity, complications, chronicity, and manageability of hypertension were not associated with its treatment. Participants who knew the normal blood pressure levels were more likely to take antihypertensive treatment than those who did not know about it (Table 3).

**Table 3:** **Association of knowledge and perception of hypertension with treatment**.

Characteristics	Categories	Treatment		Total (%)	*P* value
		Yes (%)	No (%)		
Awareness on normal BP level	Yes	56 (87.5)	8 (12.5)	64	0.02
	No	37 (68.5)	17 (31.5)	54	
Perceived severity	Yes	77 (78.6)	21 (21.4)	98	0.89
	No	16 (80.0)	4 (20.0)	20	
Perceived complication	Yes	73 (77.7)	21 (22.3)	94	0.54
	No	20 (83.3)	4 (16.7)	24	
Perceived chronicity	Yes	58 (80.6)	14 (19.4)	72	0.56
	No	35 (76.1)	11 (23.9)	46	
Perceived effect of lifestyle modification	Yes	85 (79.4)	22 (20.6)	107	0.60
	No	8 (72.7)	3 (27.3)	11	

Questions asked to measure each of the above variables – Awareness on normal BP level, “Do you know the normal blood pressure level?”; Perceived complication, “Do you think high blood pressure will have long-term health complications?”; Perceived chronicity, “Do you think that high blood pressure is a life-long disease?”; Perceived effect of lifestyle modification, “Can changing lifestyle help to lower high blood pressure?

#### Barriers to Treatment of Hypertension

More than one-fifth of the participants (25 of 118) were not under treatment. Reason behind avoiding treatment was a perception that once medicine was taken that should be continued lifelong (16 of 25). Similarly, experiencing unwanted effect or fear of long-term effect of medicines were the other reasons behind quitting the medications (8 of 25).

In FGD, the majority of the participants did not like the need of long-term use of anti-hypertensive medication and indicated it as a main barrier for not initiating the treatment. “*If medicine is taken once, it cannot be stopped. So, I have not started yet* … *no, I will not take medicine for hypertension in any condition*,” a FGD participant expressed his denial to take antihypertensive medicine though he was aware of severity of uncontrolled hypertension. Similarly, most of the participants were also worried about the adverse effect of medicine. Some quit medicine after experiencing unwanted effects like weakness, dizziness, headache, and abdominal pain. “*Medicine causes abdominal discomfort, burning sensation of hand and feet and cramping of limbs. So, I stopped taking medicine*” (FGD 1, male, 65 years). None of the participants raised the issues of unaffordability of anti-hypertensive drugs as a barrier in hypertension treatment.

Physicians also agreed that people had various beliefs on duration of intake of medicine and its adverse effect, which sometimes demotivated patients to continue or initiate medicine. “*I have encountered several patients who hesitated to take medicine with the fear of adverse effect*.”

Most of the FGD participants perceived exercise and diet control as an alternative to the medicine for effective blood pressure control. They believed that by strictly following the healthy behavior they could avoid medicine. Some participants even shared their unusual practices like taking cold showers every morning, drinking plenty of water kept in copper jog, giving up evening meal, etc., to control hypertension. “*I am drinking plenty of water, going on morning walks regularly, taking cold showers, and having low salt diet …* . *In my view, it is good to control pressure by lifestyle modifications rather than by taking medicine directly*” (FGD 2, male, 42 years). A few participants also reported using home and herbal remedies for blood pressure control. The herbal remedies they frequently used were drinking of juice expressed from bitter gourd, sponge gourd, bottle gourd, and aloe vera.

### Control of Hypertension

Among those 93 hypertensive participants under medication, 46 had controlled hypertension. Hypertension control had no statistically significant association with the socio-demographic characteristics like gender, age groups, educational level, ethnicity, occupation, and income (Table 2).

There was also no statistically significant association between the control of hypertension and smoking, alcohol consumption, fruit and vegetables intake, physical exercise, and presence of diabetes (Table 2).

#### Barriers to Control of Hypertension

Participants taking two or more medications separately or in combination had significantly higher hypertension control than others’ (Table 4). Similarly, the participants who adhered to the medications, continued the follow-up care regularly, and received the lifestyle modification advice from the physician had more control over hypertension. Waiting time in health centers for the check up was significantly associated with control of high blood pressure. The proportion of controlled hypertensive participants did not vary much with types of health facility (government or others) and health-care provider (cardiologist or others).

**Table 4:** **Association of treatment related factors with control of hypertension**.

Characteristics	Categories	Control of hypertension		Total	*P* value
		Yes (%)	No (%)		
Antihypertensive medicine	Mono-therapy	35 (44.9)	43 (55.1)	78	0.04
	Combination or Two and more medicines	11 (73.3)	4 (26.7)	15	
Access to health facility	<20 min	16 (51.6)	15 (48.4)	31	0.70
	≥20 min	27 (47.4)	30 (52.6)	57	
Waiting time in health facility	<20 min	22 (66.7)	11 (33.3)	33	0.03
	≥20 min	22 (42.3)	30 (57.7)	52	
Adherence to medication	High	30 (78.9)	8 (21.1)	38	<0.01
	Medium	16 (30.8)	36 (69.2)	52	
	Low	0 (0)	3 (100.0)	3	
Types of health-care providers	Cardiologist	30 (55.6)	24 (44.4)	54	0.17
	Others	16 (41.0)	23 (59.0)	39	
Types of health facilities	Government	12 (44.4)	15 (55.6)	27	0.54
	Other	34 (51.5)	32 (48.5)	66	
Follow-up continuation	Yes	38 (60.3)	25 (39.7)	63	<0.01
	No	7 (24.1)	22 (75.9)	29	
Satisfaction with service provider	Yes	43 (52.4)	39 (47.6)	82	0.18
	No	3 (27.3)	8 (72.7)	11	
Health-care provider advice on lifestyle modification	Yes	29 (61.7)	18 (38.3)	47	0.03
	No	17 (37.0)	29 (63.0)	46	

Non-adherence to medication was also a common problem observed among FGD participants who were under treatment. Some stopped using medicine thinking that they had completely recovered from the illness, and some were reducing the dose themselves when they felt well. “*I am not taking medicine. If my pressure crosses the limit, then only I will take medicine. I discontinued using medicine since my pressure lowered down to normal*” (FGD 2, male, 36 years). In IDI, physicians underlined the treatment non-adherence as one of the main barriers in hypertension control. “*The main reason behind uncontrolled hypertension is non-adherence to medication*.”

Lost to follow-up was another pertinent issue identified among the uncontrolled hypertensive participants. The majority of FGD participants reported that they were not on a regular follow-up for having their blood pressure checked up. They either neglected it or did not get any clear message about the need of follow-up from health-care providers. “*I have not gone for follow-up. I am following the same regimen from last one and a half years. My doctor told me to visit him only if I had problems*” (FGD 2, female, 55 years). Apart from that, participants living relatively far (1 h by bus) from the hospital did not like to visit doctor regularly. The majority of the FGD participants also reported that they did not receive any proper messages on drug use and lifestyle modification from the physicians or from health centers. Most of them complained that physician did not provide sufficient time to listen to them. Both key informants also agreed upon the issue raised by the participants and said it was because of time constraint. “*Clinicians are mostly overwhelmed by the large number of patients. They could not pay enough attention to their patients*.” They also indicated that because of lack of clear national guidelines, there was no consensus in selecting both pharmacological and non-pharmacological interventions for the management of hypertension in Nepal. “*We do not have any national guidelines on hypertension treatment. That brings inconsistency and lack of uniformity among physicians while selecting the type of anti-hypertensive medicine, dosage and non-pharmacological interventions*.”

## Discussion

Study findings suggested that diagnosed but untreated and treated but uncontrolled hypertension occur largely in the study population. Treatment status varied significantly with age groups, ethnicity, occupation, and household income. Long-term treatment need and perceived side effects of anti-hypertensive medicines were the major barriers in treatment of hypertension. Similarly, intake of single antihypertensive medicine, long waiting time at health facility, non-adherence to medication, dis-continuation of follow-up care, lack of uniformity in hypertension treatment, and failing to deliver the lifestyle modification messages from health-care providers were the major barriers in control of hypertension. Small sample size and the restriction of FGDs to controlled hypertensive participants were the concerning limitations of the study.

Our study found that 78.8% (93 of 118) of aware hypertensive participants were under anti-hypertensive medication and 49.4% (46 of 93) of the participants who took medicine had controlled blood pressure. The findings were higher than the results of a study conducted in similar setting in Kathmandu in 2006, which revealed that hypertension treatment rate among aware hypertensive participants was 63.3% and hypertension control rate among the participants on medication 22.8% (29). The result of a similar study conducted in urban slum in India reported higher percentage of hypertension control (52.9%) than ours (30). The proportion of participants having controlled blood pressure among those receiving treatment was comparable to the prevalence of hypertension control reported in high-income country (40.7%), low-income country (40.2%), upper–middle-income country (32.3%) and low–middle-income country (26.9%) (4). However, these findings were based on study conducted among hypertensive participants as a whole between January 2003 and December 2009.

Our study showed that participants intentionally avoided treatment because of a long-term need of medication and a fear of side effects. Similar findings were reported from various studies conducted in Canada, Netherlands, and Thailand. They reported a fear of long-term problems from taking drugs (13). A metaanalysis synthesized the findings that people had widespread caution about taking medicines. They feared drug dependency and tolerance and the potential harm from long-term medication (8). In our study, some participants who were reluctant to take medicine showed considerable preference to non-pharmacological measures like home remedies or lifestyle modification instead. A Chilean study also found that 8.5% of patients in their primary care-based cohort were managed exclusively with other than medicine (31). None of the participants reported the medical care expenses as a barrier in hypertension treatment and follow-up care. This result should cautiously be generalized to the other parts of the country where the majority of people has comparatively less purchasing capacity than the people living in municipalities of Kathmandu (32).

The use of two or more medications combinedly or separately was associated with hypertension control. Similar to our finding, a meta-analysis conducted among 42 trials concluded that combination therapy (two different classes) gave five times better result in controlling blood pressure than doubling the dose of one drug (33). Evidence from Pakistan suggests that the prescription of optimal medications also contributes significantly to the better adherence of medication (10). Even though use of combination or multidrug therapy had better result in controlling hypertension, a large percentage of participants (83.8%) in our study were receiving mono-therapy for the treatment of hypertension. That was contrasted with a study conducted outside Nepal where the majority of the patients (57.1%) used combination of drugs (31). Our study found that calcium channel blocker was the most frequently used medicine among the study participants. Even though the ongoing free health-care policy endorsed by Nepal government listed calcium channel blocker as a freely available essential anti-hypertensive drug at primary health-care centers, insufficient supply of free essential drugs is another part of discussion and out of scope of the study (34). Similarly, very nominal number of participants (2 out of 93) visited primary health-care centers for the treatment of hypertension and none of them were receiving free medication from the centers. This suggests a major gap in hypertension treatment and application of free anti-hypertensive drug policy in Nepal. Similarly, our study showed the participants who were adhered to the medications had more control over hypertension. Non-adherence to medication is directly associated with poor outcome of disease (9, 35). Participants mainly liked to adjust the dose – by either increasing or decreasing the dosage of medication – based on the severity of symptoms they felt. Similar practice of avoiding or reducing the dose of medication deliberately was also observed among the Dutch, British, American, Brazilian, and Thai. They chose to reduce the dosage of medicine thinking that their blood pressure was controlled (13).

Lost to follow-up was another barrier seen among the uncontrolled hypertensive participants. Participants who continued the follow-up care to the health-care providers regularly had better control of hypertension than those who did not. Timely maintaining the follow-up visit ensures the good compliance over medication, thus controlling hypertension (36, 37). In Malaysia, patients are reluctant to visit physicians for the second time because of their denial of their condition, or, having failed to follow the lifestyle advice on diet and exercise, or low health literacy given by physicians (15). But, in our study, the majority of the participants missed the follow-up date because of lack of proper information about follow-up need from health-care providers.

Our study found that the participants who received the proper counseling in life-style modification and drug use had high control of blood pressure. Key informants and participants reported that health-care providers spent very insufficient time to engage with patients because of time constraint. Evidence suggests that the communication barriers between heath-care providers and patients impede effective control of hypertension (38). Moreover, regular follow-up care also leads to high compliance over treatment. A randomized controlled trial conducted in Pakistan found that relative adherence in people seeking care from general practitioners who were trained in scheduling follow-up visits, providing satisfactory consultation sessions with explanation of non/pharmacological interventions was 50% higher than in those consulting untrained general practitioners (39). Adequate counseling of patients on treatment plan, medications, and lifestyle interventions can play a substantial role in building trust between physician and patient and lead to treatment adherence and hypertension control (12). Researches synthesized the results of 27 trials and concluded that intensive combined lifestyle counseling applied for 12 to 24 months reduced blood pressure level (14). However, a Chinese study conducted among 556 hypertensive patients found that dietary counseling was ineffective to control hypertension (40).

Our study also found some system-related barrier like long waiting time in hospital and distantly located health-care facilities that demotivated people to go for a regular follow-up. A study conducted in Africa also echoed the same issues of inconveniently located health-care facilities and long waiting times within those facilities as the barriers in high blood pressure prevention and control (11). Similarly, physician’s unawareness or non-compliance over hypertension treatment guidelines was another pertinent issue that rose in the study could be a physician-related barrier in treatment and control of hypertension (6).

## Conclusion

Overall, this study explored the barriers in hypertension treatment and control that were related to persons with hypertension, health-care providers, and health system in Kathmandu, Nepal. Findings have implication on effectively addressing barriers embedded in hypertension treatment and control in Nepali urban population through designing multi-faceted strategies including health education for increasing hypertension-related health literacy in the community, issuing national guidelines for hypertension treatment, and introducing policy to overcome institutional hurdles.

## Author Contributions

SD, RD, and AP provided concept; designed and executed the study; analyzed and interpreted the data; and prepared the first draft of the manuscript. BB and SP provided an input on data collection, analysis, and interpretation of study. KT and RG contributed to study concept, design, and interpretation. All authors read and approved the final manuscript.

## Conflict of Interest Statement

The authors declare that the research was conducted in the absence of any commercial or financial relationships that could be construed as a potential conflict of interest.
